# The future of equitable science publishing

**DOI:** 10.4102/jcmsa.v1i1.50

**Published:** 2023-11-28

**Authors:** Sam Nightingale

**Affiliations:** 1 Neuroscience Institute, University of Cape Town, Cape Town, South Africa

For many years, research was hidden behind subscriptions and paywalls. This system created vast profits for the publishing houses amounting to billions of dollars per year.^1^ In our setting, there were echoes of colonialism, given that African researchers using public funds gave the fruit of their toils to wealthy global north publishers, who sold it back to them. The system was untenable.

In 2018, after many years of wrangling and protest, a dramatic intervention was announced. Plan S – the result of an international coalition representing many major research intuitions – was designed to change this system once and for all. Plan S insisted that all scientific information be published open access, namely available freely without subscription or paywall.^2^

Despite its noble aims, I would argue that Plan S has not improved things and may have inadvertently made the system worse. The vast profits made by the publishers remain, this time accrued through ‘article processing fees’ (APCs), paid by authors before their work can appear in the journal. While some level of fee might be reasonable to cover formatting and typesetting costs and coordinate the peer review, APCs are typically several thousands of dollars. In some southern African countries where libraries cannot help cover costs, even the lowest APCs can exceed the monthly salary of the researchers submitting the work, rendering them unaffordable to most.^3^ Although some publishers offer fee waiver systems for some low-resource countries, this does not fix the underlying issue of an unaffordable and potentially exploitative system.

Worse still, the APC price goes up dramatically for higher impact journals, effectively pricing researchers in low-resource settings out of the global research conversation. Our University of Cape Town (UCT) library can afford to pay a limited number of APCs up to around $1000.00, relegating our researchers to the lowest impact journals and sustaining the dominance of wealthy research institutions in the global north.

In an attempt to combat this, non-profit publishing was established. However, this has failed to reduce APCs to levels affordable for most low-resource settings. Public Library of Science (PLOS) – perhaps the most well-established non-profit publisher – charges APCs around $2000.00 – $6000.00. It is difficult to understand how this can represent an efficient and cost-effective system, particularly given that researchers typically provide their peer review and editorial support for free. The problem may stem from the absence of a functioning marketplace within science publishing. As researchers tend to select a journal based on its prestige rather than its price, the costs are not driven down.

This is where journals such as *Journal of the Colleges of Medicine of South Africa* (JCMSA) are different. The JCMSA articles are published open access with no APCs, with costs absorbed by the journal using funds raised separately. Not only does this render the system affordable to researchers in low-resource settings but it also has the interesting side effect of creating a functioning marketplace. The JCMSA pays a publisher a fee per article for typesetting and formatting and coordinating peer review. If next year a different publisher comes along offering the same service for $100.00 less per article, JCMSA can change provider to reduce cost. If the following year another publisher offers a higher quality or faster service for the same price, JCMSA could change provider to improve quality. Indeed, if an innovative tech entrepreneur finds a way to offer the same quality of service for a fraction of the cost using open-source publishing software and post-publication peer review,^4^ then JCMSA could consider it. Market forces drive innovation such that price approaches cost and quality is maximised.

Some free services such as this already exist, notably Wellcome Open Research and Open Research Africa who waive APCs for members of affiliated institutions. However, to date some researchers have been reluctant to publish their best research in these journals, fearing they will lose the prestige and the career benefits associated with a publication in high-impact journals.

In my opinion, the only way to create behaviour change among researchers towards the widespread adoption of an equitable publishing system is to remove the prestige associated with publishing in higher-impact journals. Systems for hiring, promotion and grant funding should be changed such that the quality and impact of a researcher’s output is not judged by the journals they have published in. This is not a novel or controversial assertion; the DORA declaration – which has been signed by many of the world’s major institutions including UCT – expressly states that we should move away from journal impact factors towards article-level metrics.^5^ However, no such alternative system has been widely adopted and we continue to use the journal as a surrogate measure of quality. If we can move away from this towards a better system of validation, then open access platforms without APCs, such as JCMSA, will be adopted for all levels of research output. This represents an exciting opportunity for change towards a genuinely equitable system of science dissemination.
